# Changes in hippocampal AMPA receptors and cognitive impairments in chronic ketamine addiction models: another understanding of ketamine CNS toxicity

**DOI:** 10.1038/srep38771

**Published:** 2016-12-09

**Authors:** Runtao Ding, Yanning Li, Ao Du, Hao Yu, Bolin He, Ruipeng Shen, Jichuan Zhou, Lu Li, Wen Cui, Guohua Zhang, Yan Lu, Xu Wu

**Affiliations:** 1 Department of Forensic Pathology, School of Forensic Medicine, China Medical University, No. 77 Puhe Road, Shenyang North New Area, Shenyang 110122, P. R. China; 2 Department of Forensic and Medical Laboratory, Jining Medical University, 16 Hehua Road, Jining 272067, P. R. China; 3 Center of Forensic Science Jining Medical University, Jining Medical University, 16 Hehua Road, Jining 272067, P. R. China; 4 Key Laboratory of Health Ministry in Congenital Malformation, the Affiliated Shengjing Hospital of China Medical University, Shenyang 110004, P. R. China; 5 School of Medicine and Life Science, University of Jinan-Shandong Academy of Medical Science, Jinan, Shandong 250062, P. R. China

## Abstract

Ketamine has been reported to impair human cognitive function as a recreational drug of abuse. However, chronic effects of ketamine on central nervous system need to be further explored. We set out to establish chronic ketamine addiction models by giving mice a three or six month course of daily intraperitoneal injections of ketamine, then examined whether long-term ketamine administration induced cognition deficits and changed hippocampal post-synaptic protein expression in adult mice. Behavior tests results showed that mice exhibited dose- and time-dependent learning and memory deficits after long-term ketamine administration. Western blot results showed levels of GluA1, p-S845 and p-S831 proteins demonstrated significant decline with ketamine 60 mg/kg until six months administration paradigm. But levels of p-S845 and p-S831 proteins exhibited obvious increase with ketamine 60 mg/kg three months administration paradigm. NR1 protein levels significantly decrease with ketamine 60 mg/kg three and six months administration paradigm. Our results indicate that reduced expression levels and decreased phosphorylation levels of hippocampal post-synaptic membrane GluA1- containing AMPA receptors maybe involved in cognition impairment after long-term ketamine administration. These findings provide further evidence for the cognitive damage of chronic ketamine addiction as a recreational drug.

Ketamine, a dissociative anesthetic of phencyclidine (PCP) is a non-competitive N-methyl-D- aspartate (NMDA) receptor antagonist. It has become a recreational drug, with its use spreading around the world in the last decades^1^,^2^,^3^,^4^,^5^. NMDA receptors are intimately involved in regulating synaptic plasticity and memory function^6^. As a non-competitive NMDA receptor antagonist, ketamine, either acute or chronic, can induce various cognitive impairments^7^,^8^,^9^.

Focusing on the acute effects of ketamine, some studies indicate that sub anesthetic doses of ketamine induced learning and memory impairment in developing rodents^10^,^11^. Other studies claim that acute anesthetic doses of ketamine did not affect spatial memory in adult mice, and that ketamine counteracted repetitive mechanical stress-induced learning and memory impairment in developing mice^10^,^12^.

There are few studies devoted to the long-term effects of ketamine addiction on cognitive functions. One study found ICR mice exposed to chronic sub anesthetic doses of ketamine (30 mg/kg) exhibited learning and memory deficits^13^. Yeung *et al*., found that mice exposed to sub anesthetic dose of ketamine (30 mg/kg) for six months showed a hyper-phosphorylation of tau and apoptosis in prefrontal and entorhinal cortex, indicating impairment similar to the Alzheimer’s disease^14^. However, another study showed that the same dose of ketamine administrated for six months did not show cognitive impairment compared with the control in first, third or sixth month^15^.

In the past few decades, it has been thought that ketamine-induced cognitive impairments were due to the drug’s antagonist action at the NMDA receptor family^16^,^17^, as the overwhelming major composition of NMDA receptor complexes, NMDA receptor NR1 subunit (NR1) has been reported to played an intimate role in rodents’ learning and memory process^18^,^19^,^20^. However, the accepted mechanism of cognitive damage or CNS toxicity of chronic ketamine addiction still needs to be further explored.

As excitatory synapses, AMPA receptor (composed by four subunits GluA1, GluA2, GluA3 and GluA4) triggers long-term potentiation (LTP) and long-term depression (LTD), which are fundamental for rodent hippocampal learning and memory^21^,^22^. Recent studies have suggested that hippocampal post-synaptic membrane GluA1 levels are critical for rodent learning and memory, and rodent cognitive behaviors were accompanied by hippocampal GluA1-containing AMPA receptor trafficking^23^,^24^. Mice lacking the gene encoding the GluA1 subunit exhibited LTP induction deficiency, and showed impaired hippocampal-dependent spatial memory^25^,^26^,^27^,^28^. S831 and S845 are two important serine phosphorylation sites on the GluA1 subunit intracellular carboxy terminus^29^,^30^. Both of these sites play an essential role in post-synaptic AMPA receptor regulation and synaptic plasticity^23^. S831, a site phosphorylated by protein kinase C (PKC) and calcium/calmodulin kinase II (CaMKII)^29^,^30^,^31^, upregulated phosphorylation levels of GluA1 in LTP and other cognitive behavior training^23^,^32^. Subsequently, activity-dependent synaptic trafficking of GluA1 was shown to depend on S845, a protein kinase A (PKA) phosphorylation site^33^.

In this study, we set out to establish mice chronic ketamine addiction models utilizing three and six month sub anesthetic ketamine administration, and to investigate whether mice exhibit learning and memory impairments in this model. We also investigated hippocampal post-synaptic membrane GluA1, p-S845 and p-S831 protein levels. We attempted to determine the underlying relationship between behavior performance and relevant proteins levels by analyzing the changes, with the goal of better understanding of the cognitive toxicity of this recreational drug after chronic use.

## Results

### Mice showed a spatial reference memory deficit after six months of ketamine administration

To investigate the learning and memory effects of long term ketamine administration, 30 mg/kg, 60 mg/kg ketamine and a corresponding volume of physiological saline were administrated to mice for three or six months. All mice, regardless of their corresponding treatments, displayed different degrees of decrease in escape latency to reach the hidden platform during the five day test.

**Figure 1:**
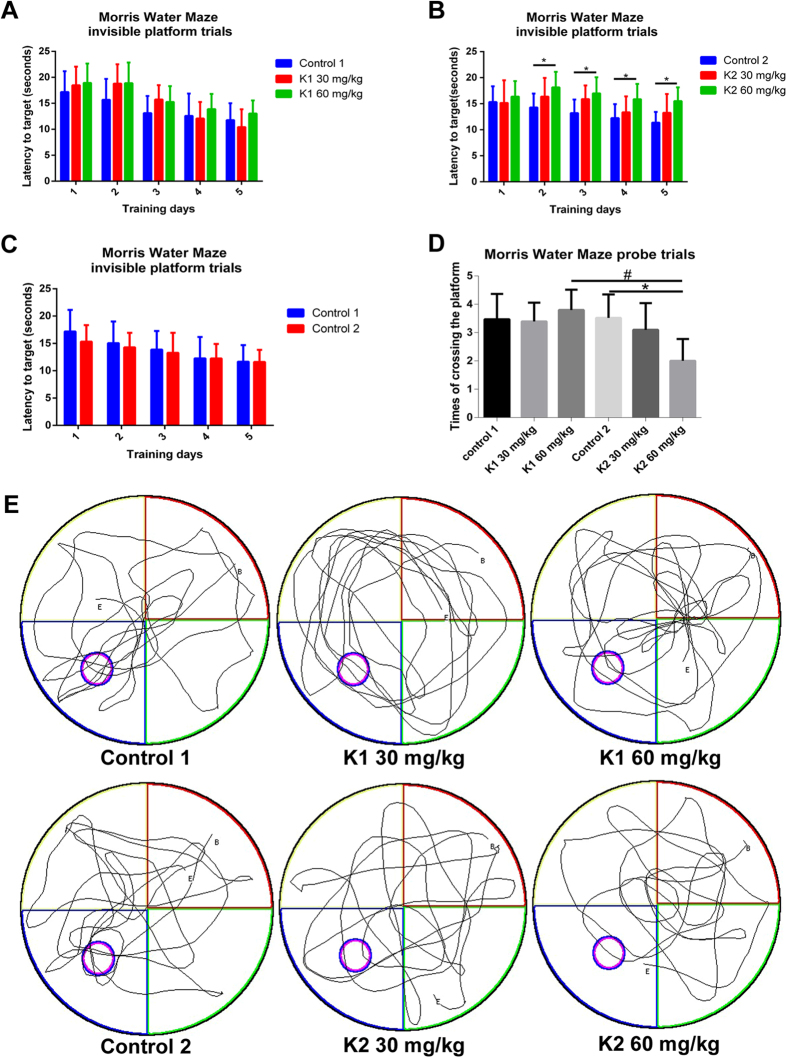
Spatial reference memory performance of mice in Morris Water Maze tests following three and six months of ketamine administration with different doses of ketamine 30 mg/kg and 60 mg/kg. (**A**) Mice performance of latency time to reach the target in Morris Water Maze invisible platform trails after three months of administration paradigm. (**B**) Significant increase of latency time to reach the target in Morris Water Maze invisible platform trails in K2 60 mg/kg in the training day 2, 3, 4, 5 after six months of administration paradigm (*p < 0.05). (**C**) Comparison of latency time to reach the target in Morris Water Maze invisible platform trails between Control 1 and Control 2. (**D**) Significant decline of the times of crossing the former platform location was observed in K2 60 mg/kg comparing with K1 60 mg/kg and Control 2 (^#^*p* < 0.05, *p < 0.05). (**E**) Representative searching swimming tracks by mice with different treatments in the probe trial test. Control 1 n = 26, K1 30 mg/kg n = 24, K1 60 mg/kg n = 25, Control 2 n = 26, K2 30 mg/kg n = 24, K2 60 mg/kg n = 25.

No significance was observed in three groups after three months of ketamine administration during five day invisible trials (Fig. 1A). Remarkably, a significant increase in latency time to target was seen in K2 60 mg/kg group on day 2, 3, 4 and 5 after six months of administration paradigm (Fig. 1B; *p < 0.05). Furthermore, we analyzed the data for both control groups to check if age (as Control 1 together with K1 30 mg/kg and K1 60 mg/kg received administration paradigm for three months but Control 2 together with K2 30 mg/kg and K2 60 mg/kg for six months) had an influence on spatial reference memory of mice in this study. Our data showed no difference between Control 1 and Control 2 groups (Fig. 1C). Mice were tested for spatial memory retention by probe trial tests 24 h after the last invisible platform trial. No significant differences were observed in groups after three months of ketamine administration (Fig. 1D). However, after six months of ketamine administration, mice in the K2 60 mg/kg group exhibited an obvious reduction in the times of crossing the former platform location (Fig. 1D; *p < 0.05). Meanwhile, mice receiving ketamine 60 mg/kg consecutive administration for 6 months exhibited a significant decline in the times of crossing the former platform location compared with the three month group (Fig. 1D; ^#^*p* < 0.05). Consistent with this, behavioral tracking results revealed that mice treated by ketamine 60 mg/kg for six months (K2 60 mg/kg) displayed less exploration in the former target location and in the target quadrant than mice treated by physiological saline for six months (Control 2) or ketamine 60 mg/kg for three months (K1 60 mg/kg) (Fig. 1E). The data from Morris Water Maze test indicated a spatial reference memory deficit after ketamine 60 mg/kg six months administration paradigm.

### Mice showed spatial working memory deficit

**Figure 2:**
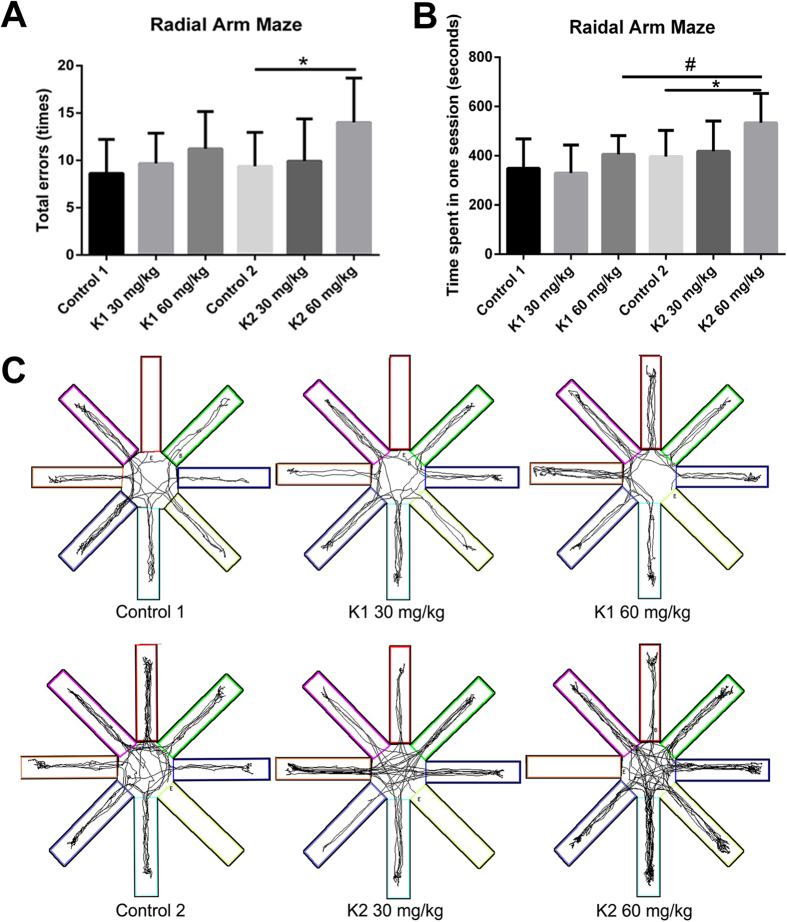
Spatial working memory performance of mice in Radial Arm Maze tests following three and six months of ketamine administration with different doses of ketamine 30 mg/kg and 60 mg/kg. (**A**) Significant increase of total errors were seen in K2 60 mg/kg comparing with Control 2 (*p < 0.05). (**B**) Significant increase of time spent in one session to accomplish a Radial Arm Maze test in K2 60 mg/kg comparing with Control 2 and K2 60 mg/kg comparing with K1 60 mg/kg (*p < 0.05, ^#^*p* < 0.05). (**C**) Representative searching tracks of mice with different treatments in the Radial Arm Maze tests acquisition trails. Control 1 n = 23, K1 30 mg/kg n = 24, K1 60 mg/kg n = 25, Control 2 n = 23, K2 30 mg/kg n = 24, K2 60 mg/kg n = 25.

We used the Radial Arm Maze test to determine the effects of long term ketamine administration on spatial working memory. Results showed that the K2 60 mg/kg group had a significant increase in total errors compared to the Control 2 group (Fig. 2A; *p < 0.05). No significant differences were observed between Control 1, K1 30 mg/kg or K1 60 mg/kg groups after the three month administration paradigm (Fig. 2A). Mice treated by ketamine 60 mg/kg for six months (K2 60 mg/kg) spent more time to accomplish a test during a session than the Control 2 group and the group that only had three months of daily administration of the same dose (Fig. 2B; *p < 0.05, ^#^*p* < 0.05). No significant differences were observed in groups of three months administration paradigm (Control 1, K1 30 mg/kg or K1 60 mg/kg) or six months administration paradigm (Control 2, K2 30 mg/kg or K2 60 mg/kg) (Fig. 2B). No difference was observed between the two control groups (Fig. 2A and B). As is shown by searching tracks of mice, behavioral tracking results indicated that mice treated by ketamine 60 mg/kg for six months (K2 60 mg/kg) displayed more entries to arms and greater locomotion density than mice treated by ketamine 60 mg/kg for three months (K1 60 mg/kg) (Fig. 1C). The Radial Arm Maze test results indicated a spatial working memory deficit after ketamine 60 mg/kg six months administration paradigm.

### Changes in hippocampal GluA1, p-S845 and p-S831 protein levels after three months of ketamine administration

**Figure 3:**
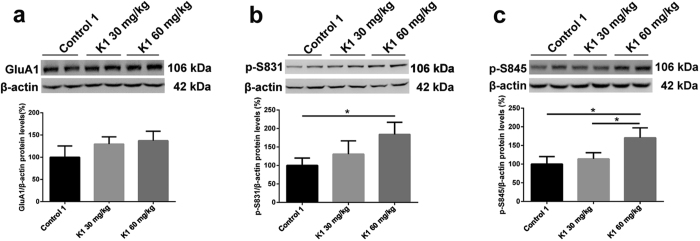
Changes of hippocampal membrane GluA1, p-S845 and p-S831 protein levels after three months of ketamine administration in the groups of Control 1, K1 30 mg/kg and K1 60 mg/kg as reveled by Western blot. (**a**) Changes of hippocampal membrane GluA1 after three months of administration paradigm in three groups. (**b**) Significant increase of p-S831 expression levels were seen in the group of K1 60 mg/kg comparing with Control 1 (*p < 0.05). (**c**) Significant increase of p-S845 expression levels were seen in the group of K1 60 mg/kg comparing with control 1 and K1 30 mg/kg (*p < 0.05).

GluA1, one of the subunits of AMPA receptor and its intracellular phosphorylation sites (S845 and S831) play a key role in hippocampal activity-dependent synaptic trafficking of AMPA receptor, which is of vital importance for learning and memory^34^,^35^,^36^. We determined the hippocampal post-synaptic membrane protein levels of GluA1, p-S845 and p-S831. Our western blot results revealed no significant changes in protein levels of GluA1, using β-actin as an internal control (Fig. 3a). However, there was a significant increase in the protein levels of p-S831 in K1 60 mg/kg group comparing with Control 1 group and a remarkable increase in the protein levels of p-S845 in K1 60 mg/kg group comparing with Control 1 and K1 30 mg/kg group (Fig. 3b,c; *p < 0.05). These results indicate that mice exposed to ketamine 60 mg/kg for three months exhibited significant increasing phosphorylation levels of hippocampal post-synaptic membrane GluA1 sites S845 and S831 without any changes in the overall expression of GluA1; three months treatment with 30 mg/kg ketamine had no effect on levels of phosphorylated S845 or S831.

### Changes in hippocampal GluA1, p-S845 and p-S831 protein levels after six months of ketamine administration

**Figure 4:**
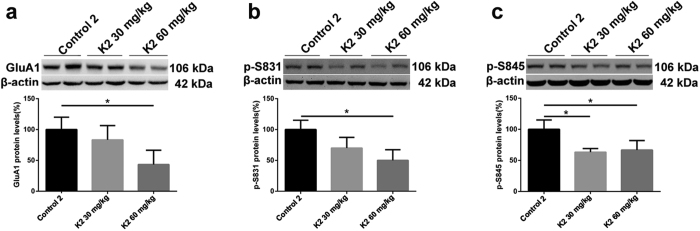
Changes of hippocampal membrane GluA1, p-S845 and p-S831 protein levels after six months of ketamine administration in the groups of Control 2, K2 30 mg/kg and K2 60 mg/kg as reveled by Western blot. (**a**) Significant decline of hippocampal membrane GluA1 expression levels were observed in K2 60 mg/kg comparing with Control 2 (*p < 0.05). (**b**) Significant decline of p-S831 were seen in K2 60 mg/kg comparing with Control 2 (*p < 0.05). (**c**) Significant decrease of p-S845 expression levels were seen in K2 30 mg/kg and K2 60 mg/kg comparing with Control 2 (*p < 0.05).

We further investigated hippocampal GluA1, p-S845 and p-S831 protein levels after six months of ketamine administration. K2 60 mg/kg group showed a significant decline in GluA1 protein levels (Fig. 4a; *p* < 0.05), using β-actin as an internal control. There was a remarkable decrease in p-S845 and p-S831 protein levels in K2 60 mg/kg group (Fig. 4b,c; *p* < 0.05). Additionally, there was an obvious decline in p-S845 levels in K2 30 mg/kg group (Fig. 4c; *p* < 0.05). The data revealed that mice exposed to ketamine 60 mg/kg for six months exhibited a significant decline of hippocampal post-synaptic membrane GluA1 expression together with decreasing phosphorylation levels in p-S845 and p-S831. This apparent decline in p-S845 protein levels was also observed after 6 months of 30 mg/kg ketamine administration.

### Change in hippocampal NR1 protein levels after three and six months of ketamine administration

**Figure 5:**
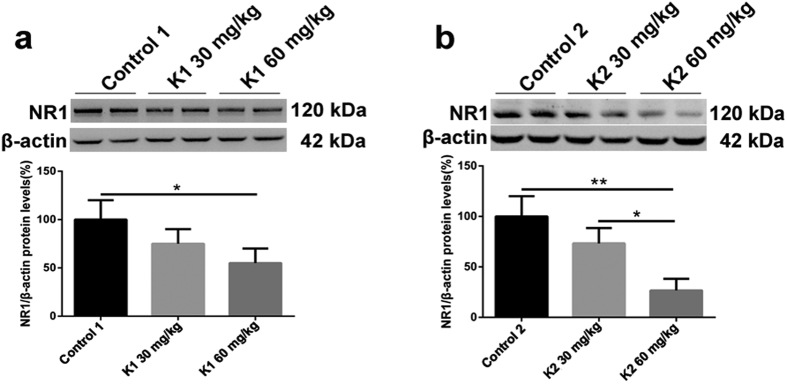
Changes of the hippocampal membrane NR1 protein levels after three and six months of ketamine administration as reveled by Western blot. (**a**) Significant decline of hippocampal membrane NR1 protein levels were observed in K1 60 mg/kg after three months of administration paradigm comparing with the group of Control 1 (*p < 0.05). (**b**) Significant decline of NR1 protein levels were seen in the group of K2 60 mg/kg comparing with the group of Control 2 and K2 30 mg/kg respectively (*p < 0.05, **p < 0.01).

NR1 is essential for NMDA receptors and composes the overwhelming majority of NMDA receptor complexes^18^. NR1 has been reported to play an intimate role in learning and memory process^19^,^20^,^37^. We investigated hippocampal NR1 protein levels with Western blots. A significant decline in NR1 protein levels was observed in K1 60 mg/kg and K2 60 mg/kg groups (Fig. 5; *p < 0.05, **p < 0.01). The K2 60 mg/kg also had a significantly lower level of NR1 when compared with the K2 30 mg/kg group (Fig. 5b; *p < 0.05).

## Discussion

Our findings from long term ketamine administration models indicate that chronic ketamine addiction induced cognitive impairments and may significantly alter these hippocampal post-synaptic membrane proteins that are of vital importance to cognitive function. We found that mice showed dose- and time-dependent learning and memory impairments after long-term sub anesthetic ketamine administration.

Learning and memory impairments after long term ketamine abuse have been reported in humans^2^,^7^,^9^. One research indicated that mice exposed to ketamine 30 mg/kg for six months expressed a ketamine toxicity, including neurodegeneration and hyperphosphorylated of tau proteins, which are similar to the Alzheimer’s disease^14^. However, according to another study, mice exposed to the same dosing paradigm showed no deficits in learning and memory^15^. We hypothesized that adult mice may express learning and memory deficits with a higher concentration of long term ketamine administration, as a previous study found that adolescent rats exposed to ketamine 80 mg/kg exhibited learning and memory impairments in Morris water maze tests, rather than at the lower dose of 30 mg/kg, in a seven day administration paradigm^38^. Consistent with our hypothesis, the higher ketamine concentration used in this study did result in a significant spatial learning and memory impairment after six months administration instead of the three month paradigm. Meanwhile, ketamine at 30 mg/kg did not induce any obvious learning and memory deficits after three or six months of administration paradigm. These results indicate that learning and memory impairments after long term ketamine administration are dose-dependent in adult mice.

According to the behavioral performances, mice exposed to ketamine 60 mg/kg did not exhibit any spatial memory impairments until after six months of administration paradigm. We further analyzed the behavioral performance of animals exposed to the same dose at different time points. In the Radial Arm Maze test, mice exposed to 60 mg/kg ketamine showed spatial memory deficits after six months treatment, but not after three months. In Morris Water Maze tests, mice exposed to ketamine 60 mg/kg showed spatial reference deficits after six months treatment, but not after three months. These results implied that ketamine-induced learning and memory deficits in adult mice are time-dependent. Coincidently, in the 2 months old ICR mice treated by long term ketamine administration, Yeung *et al*. found a extensive hyperphosphorylated tau positive sites in the PFC layers after administration for 6 months comparing by control group, but only several positive sites of hyperphosphorylated tau appeared in PFC after administration for 3 months comparing by control group^14^. The results from Yeung *et al*. indicated a time-dependent neurotoxicity resemble in our findings of learning and memory impairments after long term ketamine administration. Tan *et al*. reported that adolescent ICR mice exhibited learning and memory impairment both in one and three months of 30 mg/kg ketamine administration^13^. The reason we found mice a time-dependent learning and memory deficits but Tan *et al*. did not may be due to the fact that we used adult animals (2 months old) instead of adolescent mice (1 month old); since adult mice have more well-established brain structure and defenses against neurotoxic substances than the developing or immature brain. In fact, several studies have found acute or chronic ketamine administration induced learning and memory impairment in developing or adolescent rodents more than in adulthood^12^,^13^,^15^,^38^. To exclude the effect of age in our behavioral tests results, we analyzed two control groups (Control 1 with physiological saline administration for three months and Control 2 with that for six months) and found no significant difference between the two groups.

As a member of the AMPA receptor family, the importance of the subunit GluA1 and its phosphorylation sites S845 and S831 on learning and memory has been reviewed in detail^22^. The post-synaptic AMPA receptors play the most direct role, and are of vital importance in synaptic signal transmission; therefore changes of post-synaptic membrane receptor levels and learning and memory are closely related^39^,^40^,^41^. We investigated hippocampal membrane GluA1, p-S845 and p-S831 protein levels after three or six months ketamine administration. We found that mice exposed to 60 mg/kg ketamine for six months exhibited a significant decline in GluA1, p-S845 and p-S831 protein levels. The decline of GluA1, p-S845 and p-S831 protein levels were coincident with our behavioral results of cognitive deficit after six months ketamine administration. Previous studies reported that GluA1 and phosphorylation states of GluA1 Serine sites S845 and S831 are essential for hippocampal-dependent spatial memory^26^,^42^,^43^. However, we were surprised to discover that mouse hippocampal membrane p-S845 and p-S831 protein levels significantly increased with 60 mg/kg ketamine administration for three months. This phenomenon was not easy to reconcile with our behavior results showing that mice did not display learning and memory deficits after the three month ketamine administration paradigm. Under ordinary conditions, the upregulated hippocampal p-S845 and p-S831 protein levels probably indicated some kinds of improvement on learning and memory founction^22^, but there was no behavioral evidence for improved cognition after three months of ketamine administration in our experiment. As a non-competitive NMDA receptor antagonist, it is believed that ketamine induces learning and memory deficits via the NMDA receptor pathway^16^,^44^. NR1 is essential for NMDA receptors and composes the overwhelming majority of NMDA receptor complexes^18^, and plays a critical role in learning and memory processes^19^,^20^,^37^. Therefore, we investigated hippocampal membrane NR1 protein levels after ketamine administration. Our Western Blot results showed clearly that there was a significant decline in hippocampal NR1 protein levels after 60 mg/kg ketamine administration for three or six months, with a more dramatic decrease at six months.

After six months ketamine administration at a dose of 60 mg/kg, mice exhibited learning and memory deficits and significant decline in hippocampal GluA1, p-S845 and p-S831 and NR1 protein levels. The changes in hippocampal membrane protein levels and cognitive deficits were in agreement with previous studies showing that mice lacking GluA1, or with mutations at the S845 or S831 sites, or hypomorphic NR1 gene expression exhibited impaired hippocampus-dependent spatial memory^20^,^22^,^26^,^45^.

Mice exposed to 60 mg/kg of ketamine for three months did not exhibit learning and memory impairments, but did show an obvious decline in hippocampal membrane levels of NR1 and significant increase in p-S845 and p-S831 levels. Taking into consideration that numerous reports about NR1 levels decline inducing cognition impairment and p-S845, p-S831 levels increase contributing to learning and memory function, we hypothesize that the up-regulated levels of p-S845 and p-S831 may offset the impact of decreased NR1 levels, so these mice did not show learning and memory impairments. Previous studies reported that NR1 plays an irreplaceable role in learning and memory process, and mice with a “silent” NR1 expression exhibit impaired hippocampus-dependent spatial memory^20^,^37^. However, up-regulated phosphorylation levels of S845 and S831 might facilitate NMDA-dependent LTP and were shown to be essential for NMDA-dependent LTD, which are essential for learning and memory^42^,^43^. We hypothesize that changes in hippocampal post-synaptic AMPA receptors may also be involved in mouse learning and memory impairments after long-term ketamine administration, not just NMDA receptors as the previous theory described.

Our findings demonstrated that adult mice showed dose and time dependent learning and memory impairments after long-term sub anesthetic dose of ketamine administration. Mice hippocampal membrane GluA1, p-S845 and p-S831, NR1 protein levels exhibited dose and time dependent changes. It is probable that these learning and memory deficits maybe connected to changes in hippocampal GluA1 levels and the phosphorylation states of S845 and S831, not merely NMDA receptor levels. However, how these proteins act in the ketamine-induced hippocampal cognitive deficits and the mechanism of ketamine-induced hippocampal cognitive deficits still remains to be determined. PKA and CaMKII are two proteins specific targeting the GluA1 phosphorylation sites S831 and S845 respectively^43^. Studies have shown that the CaMKII and PKA signaling pathways are critical for some specific memory formation processes and for LTP conduction^29^,^46^. These studies, combined with our results, suggest that learning and memory deficits after long term ketamine administration may be associated with these downstream signaling events. Continuing to explore the underlying changes between these proteins and AMPA receptors in long term ketamine-induced cognitive deficits is critical to move forward.

In summary, our findings indicate that long term ketamine administration induced learning and memory impairment and changes of hippocampal membrane levels of GluA1, p-S845 and p-S831, and NR1 in a dose- and time- dependent manner. Based on the irreplaceable functions of these proteins in learning and memory initiation and consolidation, we expect that reduced levels and dephosphorylation of hippocampal membrane GluA1-containing AMPA receptors are involved in the cognitive deficits in chronic ketamine addiction.

## Methods

### Animals

180 naive adult (two months old) male C57/BL6 mice from Laboratory Animal Centre of China Medical University, weighing 17–22 g, were housed 3–4 per cage and maintained on a 12 h light/dark cycle (lights out at 6:00 PM). Mice had unlimited access to water and food in their home cages. The experiments with animals were approved by the Animal Research Ethics Committee of China Medical University in accordance with China experimental animal adiministrative regulations. All efforts were made to minimize the number of animals used and to reduce their suffering.

### Ketamine administration

In order to establish chronic ketamine addiction model, we gave mice a three or six month course of intraperitoneal ketamine daily administration, as described by previous studies^13^,^14^,^15^. Mice were randomly assigned to six groups, with 30 mice per group: Control 1 were given physiological saline for 3 months; K1 30 mg/kg were givein 30 mg/kg ketamine for three months; K1 60 mg/kg were given 60 mg/kg ketamine for three months; Control 2 were given physiological saline for six months; K2 30 mg/kg were given 30 mg/kg ketamine for six months; and K2 60 mg/kg were given 60 mg/kg ketamine for six months. Animals received intraperitoneal ketamine (ketamine hydrochloride, Fujian Gutian Pharmaceutical Co., Ltd, Gutian, Fujian, People’s Republic of China, dissolved in physiological saline) or equal volumes of physiological saline, for 90 or 180 consecutive days. Mice receiving the 60 mg/kg dose experienced a transient dystaxia state, whereas the 30 mg/kg dose induced activity. During the entire administration paradigm, 17 mice died (detailed death statistics: Control 1, 2 deaths; K1 30 mg/kg, 4 deaths; K1 60 mg/kg, 1 deaths; Control 2, 4 deaths; K2 30 mg/kg, 3 deaths; K2 60 mg/kg, 3 deaths). The number of deaths between each group were not statistically significant. Every corpse was autopsied when the body was found in the morning check. Four autopsies found abdominal cavity bleeding, three others saw visceral organ rupture (two hepatic ruptures and one kidney rupture), the rest found no apparent injuries or disease.

### Behavioral testing

**Figure 6:**
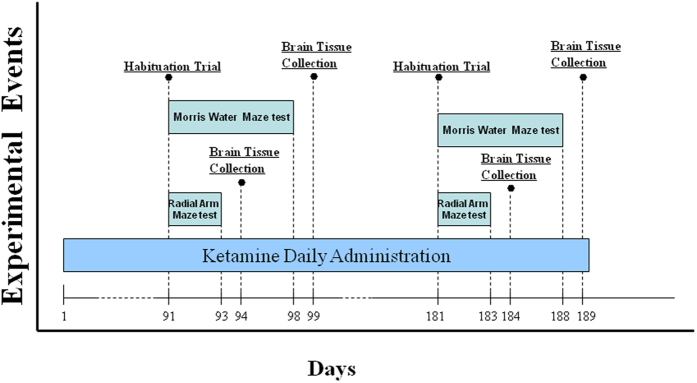
A schedule of ketamine administration and behavioral assessment. Mice received ketamine daily administration for 90 or 180 days (3 or 6 months respectively). For the three months administration paradigm, Morris Water Maze tests began on Day 91 with daily training sessions that lasted until Day on 98 of the study, Radial Arm Maze tests started on Day 91 with daily training sessions that lasted until on Day 93. For the six months administration paradigm, Morris Water Maze tests began on Day 181 and lasted until Day 188 of the study, Radial Arm Maze tests started on Day 181 and lasted until on Day 183. We surveyed the first sessions of escape latency (Day 93–97) and probe trials (Day 98) of mice on three months administration paradigm and the second sessions of escape latency (Day 183–187) and probe trials (Day 188) of mice on six months administration paradigm, to assess for spatial reference memory performance. Meanwhile, trials for the total errors and time spent to accomplish a session (Day 92–93 and Day 182–183) of mice in the Radial Arm Maze test were conducted to assess for spatial working memory performance as well. Animals were euthanized on Day 94, 99, 184 and 189 of the study, and brain tissue was dissected, collected, and stored in the −80 °C freezer.

All the behavior tests were performed 24 hours after the last intraperitoneal injection and used the SMART™ tracking software program (San Diego Instruments, San Diego, CA). All mice were in a normal state before and during the behavior acquisition. Data statistical analyses of behavior tests were performed using GraphPad Prism 6.01 software. All behavior tests were carried out according to the experimental schedule as shown in Fig. 6.

#### Morris Water Maze (MWM) test

We used the MWM to evaluate spatial learning and memory. Mice were tested in the MWM paradigm as previously described^47^. Briefly, in spatial learning tests, mice were first tested for their performance on the test with a visible platform to exclude the possibility that the mice had impairment in visual ability or in motivation, and to facilitate their habituation to the black circular water pool. Mice were trained in the water pool (100 cm diameter) for two consecutive days (4 trials per day), the water was made opaque using milk. The walls of the maze and the room displayed spatial cues, and a white plastic platform (8 cm diameter) was used, which protruded 2 cm above the surface of the water. Mice were allowed to swim freely for 60 sec, and mice landing on the platform were permitted to stay on it for 20 sec. Mice that were unable to find the visible platform were placed on the platform for an extra 10 sec. Most of the mice receiving the test exhibited good habituation and mobility.

Mice were then trained in the pool with a hidden platform for five consecutive days (3 trials per day) with spatial cues on the walls of the maze and the room. The platform was hidden 0.8 cm below the surface of the water; the water was made opaque using milk to prevent the mice from seeing the platform. The water was maintained at a temperature of 22–23 °C with a digital temperature maintaining system. The platform location was kept constant and starting points were changed in every trial to avoid track memorization. When the mouse found the platform or when 60 sec had elapsed, mice were allowed to rest on the platform for 10 sec. Latency to the target was recorded. After the completion of the five day learning phase, mice were tested for memory retention of platform location. The platform was removed and mice were allowed to swim freely for 60 sec. These probe trials were conducted 24 h after the last training trials. The entrance numbers of platform location were recorded. Data was traced by the SMART™ tracking software program. Tests were conducted under quiet conditions in order to reduce stress of mice. During the Morris water maze test, mice that exhibited passivity or thigmotaxic swimming pattern were excluded from analysis.

#### Radial arm maze test

The radial arm maze test was used to measure spatial working memory^48^,^49^. The radial arm maze (Panlab, S. L., Barcelona, Spain) was made up of an octagonal central platform (32 cm diameter) and eight equally spaced radial arms (50 cm long, 12 cm wide). Before the training sessions, food was restricted for 24 hours; body weight was controlled in the range of no less than 95% of the baseline weight. During each session, animals were allowed to visit the arms of the maze to obtain food pellets until the eight arms had been visited or 20 min had elapsed. The maze was set in an experimental room with external visual cues. The experimenter monitored the movements of the animals via the SMART™ tracking software program (Panlab, SL, Barcelona, Spain). The sum of non-visited arms and re-entry into arms was scored as a working memory error. The apparatus was cleaned with ethanol solution between trials. All procedures were performed during the light period. Mice that exhibited passivity were excluded from analysis.

### Hippocampal Membrane Protein Fractionation and Western blot

The method used to obtain post-synaptic membrane proteins from the mice hippocampi was a modification of previous procedures^50^,^51^. Briefly, hippocampi from mice were gathered, resuspended in the solution of 0.32 mol/L sucrose, 4 mmol/L 4-(2-hydroxyethyl)-1-piperazineethanesulfonic acid (pH 7.4) with protease/phosphatase inhibitors (Sigma-Aldrich, St. Louis, Missouri) and homogenized. With utilization of centrifugations, lysis, and sucrose gradient, a PSD pellet was made. Hippocampal membrane proteins from the PSD pellet were extracted using the ProteoExtract Transmembrane Protein Extraction Kit (71772, Millipore, Billerica, Massachusetts). Then 20 μg of each lysate was separated on SDS-PAGE gels and transferred to PVDF membranes (Bio-Rad, Hercules, CA) using a semi-dry electrotransfer system (Amersham Biosciences, San Francisco, CA). The blots were blocked by 4% BSA, then the blots were incubated overnight at 4 °C with primary antibodies, followed by washes and incubation with appropriate secondary antibodies, and visualized with a chemiluminescence system (Tanon 5200, Shanghai, China). Data statistical analyses of blots were performed using GraphPad Prism 6.01 software.

The following antibodies were used: anti-GluA1 (1:2000, AB1504, Millipore); anti-β-actin (1:5000, ab8226, Abcam); anti-phospho-ser831 GluA1 (1:2000, 04-823, Millipore); anti-phospho-ser845 GluA1 (1:2000, AB5804, Millipore); anti-NR1 (1:1000, ab109182, Abcam).

### Statistical analysis

Data were expressed as mean ± SEM. The number of samples varied from 9 to 28, depending on the experiment, and all sample data was normally distributed. Analysis of variance (ANOVA) with Turkey’s post hoc tests or unpaired Student t tests used to compare differences from groups. A *p* value less than 0.05 (* or #) and 0.01 (**) were considered statistically significant. The significance testing was two-tailed, and SPSS 19.0 was used to analyze the data.

## Additional Information

**How to cite this article**: Ding, R. *et al*. Changes in hippocampal AMPA receptors and cognitive impairments in chronic ketamine addiction models: another understanding of ketamine CNS toxicity. *Sci. Rep.* **6**, 38771; doi: 10.1038/srep38771 (2016).

**Publisher's note:** Springer Nature remains neutral with regard to jurisdictional claims in published maps and institutional affiliations.
